# Dried Plum’s Unique Capacity to Reverse Bone Loss and Alter Bone Metabolism in Postmenopausal Osteoporosis Model

**DOI:** 10.1371/journal.pone.0060569

**Published:** 2013-03-29

**Authors:** Elizabeth Rendina, Kelsey D. Hembree, McKale R. Davis, Denver Marlow, Stephen L. Clarke, Bernard P. Halloran, Edralin A. Lucas, Brenda J. Smith

**Affiliations:** 1 Department of Nutritional Sciences, Oklahoma State University, Stillwater, Oklahoma, United States of America; 2 Comparative Medicine Group, Kansas State University, Manhattan, Kansas, United States of America; 3 Veterans Affairs Medical Center, University of California San Francisco, San Francisco, California, United States of America; Harvard Medical School, United States Of America

## Abstract

Interest in dried plum has increased over the past decade due to its promise in restoring bone and preventing bone loss in animal models of osteoporosis. This study compared the effects of dried plum on bone to other dried fruits and further explored the potential mechanisms of action through which dried plum may exert its osteoprotective effects. Adult osteopenic ovariectomized (OVX) C57BL/6 mice were fed either a control diet or a diet supplemented with 25% (w/w) dried plum, apple, apricot, grape or mango for 8 weeks. Whole body and spine bone mineral density improved in mice consuming the dried plum, apricot and grape diets compared to the OVX control mice, but dried plum was the only fruit to have an anabolic effect on trabecular bone in the vertebra and prevent bone loss in the tibia. Restoration of biomechanical properties occurred in conjunction with the changes in trabecular bone in the spine. Compared to other dried fruits in this study, dried plum was unique in its ability to down-regulate osteoclast differentiation coincident with up-regulating osteoblast and glutathione (GPx) activity. These alterations in bone metabolism and antioxidant status compared to other dried fruits provide insight into dried plum’s unique effects on bone.

## Introduction

Bone remodeling is a dynamic process involving the orchestration of bone resorption, formation and mineralization. When the balance between these anabolic and catabolic events is altered or disrupted, skeletal quality can be compromised, resulting in an increase in fracture risk. An estimated 1.5 million osteoporotic-related fractures occur in the U.S. each year and more than 43 million Americans have been diagnosed with osteoporosis or osteopenia [1]. Furthermore, as the population demographics shift toward an older society, the prevalence of osteoporosis is projected to increase 20% by 2020 [1], [2].

To date, the treatment options approved by the Food and Drug Administration exert their effects either by decreasing bone resorption or increasing formation of bone tissue. Anti-resorptive agents, such as bisphosphonates and denosumab decrease bone loss by inhibiting the differentiation and catabolic activity of osteoclasts in part by promoting osteoclast apoptosis [3]. Alternatively, intermittent parathyroid hormone (PTH) therapy (1–34 amino acid sequence) is considered anabolic in nature as it acts to increase bone formation and mineralization by up-regulating osteoblast differentiation and activity [4]. While anti-resorptive and anabolic treatment options have had a significant impact on osteoporosis management, they can be cost prohibitive and may be accompanied with undesirable side-effects such as osteonecrosis of the jaw, gastrointestinal distress, dizziness, and leg cramping [5], [6]. Due to the projected increase in the prevalence of osteoporosis and the need for better treatment options, the search continues for alternative strategies to maintain and/ or restore skeletal health.

Post-menopausal bone loss remains the most common cause of osteoporosis [2]. During the first 5–10 years after menopause, a physiological state exists in which bone turnover (*i.e*., both resorption and formation rate) is accelerated with the catabolic activity of osteoclasts favored over the anabolic activity of osteoblasts. The classic model used to study post-menopausal bone loss has been the ovariectomized (OVX) rat and mouse. This model has provided much insight into the effects of ovarian hormones, such as estrogen, progesterone, and follicle stimulating hormone (FSH), and their direct influence on bone metabolism [7]–[9]. Aside from these more direct effects on bone cells, estrogen’s more recently recognized regulatory role in cellular inflammatory and oxidative processes has resulted in an increased interest in targeting these pathways for therapeutic purposes [10]–[12]. The detrimental effects of postmenopausal osteoporosis, along with the limited treatment options available to protect or reverse osteoporotic bone loss, has led investigators to explore the possibility of other potential treatment options.

Fruits and vegetables have been noted for a variety of health benefits including decrease in some cancer risks [13]–[18], improved cardiovascular health [19]–[23], and weight management [20], [24], [25]. Natural compounds such as those found in many fruits and vegetables exert anti-inflammatory and anti-oxidant activity that have the potential to attenuate bone loss. The up-regulation of antioxidant systems (*i.e*., glutathione peroxidase or GPx) has been shown to suppress bone resorption associated with estrogen deficiency [26]–[29]. Dried plums (*Prunus domestica L.*), which have a high polyphenolic content and oxygen radical absorbance capacity (ORAC) score, have been shown to protect against and reverse bone loss in gonadal hormone deficient rat and mouse models of osteoporosis [30]–[34]. These studies with dried plum have consistently demonstrated that dried plum improves bone mineral density (BMD), trabecular bone microarchitecture, and biomechanical properties [30]–[34]. While dried plum has a number of components that may be exerting these osteoprotective effects, both *in vitro* [35], [36] and *in vivo* (*unpublished data)* work by our laboratory suggests that the polyphenols are in large part responsible for these observations. Although the mechanism has yet to be fully described, dried plum’s ability to restore bone mass appears to be associated with an increase or maintenance of bone formation in conjunction with suppression of bone resorption. These promising findings have led to the question of whether or not other dried fruits with known anti-oxidative properties can reverse bone loss to the extent of dried plum [31], [33].

This study was designed to compare the efficacy of several dried fruits (*i.e*., dried plum, apple, apricot, grape, and mango) in the restoration of bone in an osteopenic OVX mouse model and to provide further insight into these dried fruits’ mechanism of action. The selection of dried fruits that were used in this study was based on the fruit undergoing similar processing to dried plum, being a rich source of antioxidants and/or having potential osteoprotective properties [37]–[39].

## Materials and Methods

### Animal Care and Diets

Female 12 week-old C57BL/6 mice (Charles River, Wilmington, MA) were housed in an environmentally controlled laboratory animal research facility. Following a 7-day acclimation period, mice were anesthetized using a ketamine/xylazine cocktail (80 mg ketamine and 8 mg xylazine/kg body weight) for either a sham-operation (Sham) or bilateral ovariectomy (OVX), and maintained on the control diet immediately following surgery for 2 weeks to induce bone loss. A subset of Sham and OVX animals (*n*  =  6/group) served as baseline control groups, and were sacrificed after 2 weeks to confirm bone loss by whole body and spine BMD. Once bone loss had been established, mice were assigned to one of seven treatment groups (*n*  =  8 mice/group): Sham control diet (AIN-93M), OVX control diet, or OVX mice consuming control diet supplemented with 25% (w/w) dried plum, dried apple, dried apricot, dried grape or dried mango for 8 weeks. Dried plum, apple, and apricot were provided by Mayan Sun Inc. (Yakima, Washington), while the dried grape and mango were prepared following a similar drying protocol in our laboratory. All diets were isocaloric and adjusted for macronutrient, calcium, phosphorus, and fiber content. Food intake and body weights were monitored and recorded weekly.

At the end of the study, animals were anesthetized using ketamine/xylazine, whole body dual-energy x-ray absorptiometry (DXA; LunarPIXI, GE Medical Systems, Madison, WI) scans were performed to determine whole body composition and bone density, and animals were exsanguinated via the carotid artery. A femur was cleaned of soft adhering tissue and the bone marrow was flushed with ice cold phosphate buffered saline (PBS) using a 25 mm gauge needle. Both the flushed femur and bone marrow were processed for RNA extraction. One tibia and the spine were also excised and cleaned of soft tissue. Bone densitometry of the spine was performed and both the tibia and 4^th^ lumbar vertebra were analyzed for structural alterations by micro-computerized tomography (µCT). This study was carried out in strict accordance with the recommendations in the Guide for the Care and Use of Laboratory Animals of the National Institutes of Health. The protocol was approved by the Institutional Animal Care and Use Committee of Oklahoma State University (ACUP#HE096).

### Bone Densitometry Assessment

In addition to whole body bone mineral area (BMA), content (BMC), and density (BMD), excised spines were also scanned on a plastic platform and a region of interest (ROI) was identified to include the 4^th^ and 5^th^ lumbar vertebra (i.e., L4 and L5). All DXA scans were analyzed using PIXImus Series Software version 1.4x.

### Histology of the Proximal Tibia

Excised tibias were fixed in 10% neutral buffered formalin (NBF) for 48 hours and then transferred to 70% ethanol. Tibias were then decalcified in Cal-Rite (Thermo Scientific, Richard Allan Scientific) and processed by dehydration through sequential ethanol steps, cleared with toluene, and paraffin embedded. Tibias were cut in the sagittal plane (5 µm) using a microtome (Leica RM2165, Wetzlar, Germany), and sections stained with hematoxylin and eosin.

### Microcomputed Tomography Analyses

Bone microarchitecture of the tibia and 4^th^ lumbar vertebra was assessed by micro-computed tomography (µCT40, SCANCO Medical, Switzerland). The proximal tibial metaphysis and mid-diaphysis were used to analyze trabecular and cortical bone, respectively. Scans of the tibial metaphysis were performed at a resolution of 2048×2048 pixels. Semi-automated contours, starting 60 µm distal to the proximal growth plate, included a 750 µm volume of interest (VOI) of only secondary spongiosa for analysis of trabecular bone. Trabecular parameters evaluated included bone volume expressed per unit of total volume (BV/TV), trabecular number (Tb.N.), trabecular thickness (Tb.Th.), trabecular separation (Tb.Sp.) connectivity density (ConnDens) and structural model index (SMI). Analysis of cortical bone was performed by analyzing a 120 µm section at the mid-point of the tibia. Assessment of cortical bone parameters included cortical porosity, thickness, area, and medullary area of the tibial mid-diaphysis. The acquired images were analyzed at a threshold of 300, and a sigma of 0.7 and support of 1.0 were used for both trabecular and cortical bone of the tibia.

Analyses of the spine were performed by acquiring images at a resolution of 1024×1024 pixels, 80 µm from the dorsal and caudal growth plates. Similar to the tibial analysis, semi-automated contours were placed to assess secondary spongiosa within the VOI. The images were analyzed at a threshold of 340, and a sigma and support of 1.2 and 2.0, respectively.

### Biomechanical Testing of Vertebra and Tibia with Finite Element Analysis

Finite element (FE) analysis software (SCANCO Medical) was used to evaluate alterations in biomechanical properties of the trabecular bone. Voxels from the VOI were converted into 8-node brick elements from micromechanical FE [40]. The elements in this FE model had linear, elastic and isotropic material properties. Simulated compression testing was applied on trabecular regions of the vertebral body and tibial metaphysis at a constant force. Total force, stiffness, and size-independent stiffness were determined.

### Analysis of Plasma Antioxidant Capacity

Due to the potential influence dried fruits have on oxidative status, along with the impact this may have on osteoblast and osteoclast activity, plasma glutathione peroxidase (GPx) activity was determined using a commercially available kit from Cayman Chemical (Cat#703102, Ann Arbor, MI) [26], [29], [41], [42]. All samples were diluted 1∶2 and the assay was carried out according to the manufacture’s protocol. The intra- and inter-assay coefficients for the GPx assay were 5.7% and 7.2%.

### RNA Extraction and Quantitative Real-Time PCR

Alterations in the expression of genes associated with bone metabolism and apoptosis were assessed in bone by quantitative real-time PCR (qRT-PCR). Total RNA was extracted from pulverized-flushed femurs (Spex 6770 Freezer Mill, Metuchen, NJ) and bone marrow using Trizol Reagent and following the manufacturers protocol (Life Technology, Rockville, MD, USA). The hard tissue and bone marrow specimens were assessed separately because they represent an osteoblast rich cite and a cite in which osteoclasts reside [43]. RNA from the bone marrow was further purified with a second Trizol extraction. The A_260_/A_280_ ratio was obtained using a Nanodrop Spectrophotometer (Rockland, DE) to determine quantity of RNA and gel electrophoresis was carried out to verify the quality of all RNA samples. qRT-PCR was then performed using 2 µg of total RNA pre-treated with DNase I and subjected to reverse-transcription (Superscript II, Invitrogen, Carlsbad, CA). cDNA (50 ng) was used for each qRT-PCR reaction and all reactions were assayed in duplicate using SYBR green chemistry (SABiosciences, Valencia, CA) on the Applied Biosystems 7900HT Fast Real-Time PCR System (Foster City, CA). All qRT-PCR results were evaluated by the comparative cycle number at threshold (C_T_) method (User Manual #2, Applied Biosystems), using cyclophilin b (*Cyclo*) as the invariant control (**Table 1**).

**Table 1:** List of Validated Primer Sequences used for qRT-PCR.

NCBI Gene Accession Reference	Symbol	Name	Sequence
NM_007431	*Alp*	Alkaline phosphatase	QF 5'- GGT ATG GGC GTC TCC ACA GT -3'
			QR 5'- GCC CGT GTT GTG GTG TAG CT -3'
NM_007523.2	*Bak1*	Bcl-2 homologous antagonist/killer-1	QF 5'- ACG AAC TCT TCA CCA CCA AGA TCG CCT -3'
			QR 5'- TCA AAC CAC GCT GGT AGA CGT ACA -3'
NM_001032298	*Ocn*	Osteocalcin	QF 5'- TGA GCT TAA CCC TGC TTG TGA CGA -3'
			QR 5'- AGG GCA CAG GTC CTA AAT AGT -3'
NM_009810	*Casp3*	Caspase-3	QF 5'- CAT AAG AGC ACT GGA ATG TCA TCT C -3'
			QR 5'- CCC ATG AAT GTC TCT CTG AGG TT -3'
NM_009811	*Casp9*	Caspase-9	QF 5'- CTG GGA CGC TCT GCT GAG T -3'
			QR 5'- CCA GAT CCT GCC TGC TGA AT -3'
NM_007742	*Col1a1*	Collagen Type 1	QF 5'- CGT CTG GTT TGG AGA GAG CAT -3'
			QR 5'- GGT CAG CTG GAT AGC GAC ATC -3'
NM_011149	*Cyclo*	Cyclophilin B	QF 5'- TGG AGA GCA CCA AGA CAG ACA -3'
			QR 5'- TGC CGG AGT CGA CAA TGA T -3'
NM_016791	*Nfatc1*	Nuclear factor of activated T-cells, cytoplasmic 1	QF 5'- GCG AAG CCC AAG TCT CTT TCC -3'
			QR 5'- GTA TGG ACC AGA ATG TGA -3'
NM_008764	*Opg*	Osteoprotegerin	QF 5'- TCC TGG CAC CTA CCT AAA ACA GCA -3'
			QR 5'- ACA CTG GGC TGC AAT ACA CA -3'
NM_011613	*Rankl*	Receptor activator for nuclear factor κ B ligand	QF 5'- CTG ATG AAA GGA GGG AGC AC -3'
			QR 5'- GAA GGG TTG GAC ACC TGA ATG -3'

### Statistical Analysis

Statistical analyses were accomplished using SAS Version 9.2 (SAS Institute, NC). Comparisons were made between dietary treatment groups using one-way ANOVA, followed by *post hoc* analysis with Fischer’s least square means separation test when F values were significant. Student’s paired t-test was used for comparisons made between the sham-operated (Sham) and OVX groups at baseline. The percent change in trabecular bone was calculated by determining the difference between the OVX group mean BV/TV at baseline and BV/TV of individual animals within a given treatment at the final time point, and then dividing that value by the baseline value. All data are presented as mean ± standard error (SE) and α was set at 0.05.

## Results

### Body Weight, Body Composition, and Uterine Weights

At the end of the 2 week baseline period, there were no differences in initial body weight or body composition between the Sham and OVX animals (*data not shown*), but as anticipated after the 8 week treatment period the OVX group on control diet weighed significantly more than the Sham group (**Table 2**). The OVX control animals also had greater body fat mass and percent fat, while no alterations were observed in lean mass **(** **Table 2** **)**. The dried plum, apple, and mango groups had lower body weight compared to the OVX control group and the mean body weight and body composition (*i.e*., fat mass and percent fat) of the dried plum and apple groups were similar to the Sham. Although the dried apricot and grape diets did not prevent OVX-induced weight gain, animal consuming these diets had a lower fat mass and percent fat compared to OVX animals on control diet **(** **Table 2** **)**. It is important to note that these body weight and body composition changes occurred despite no difference in food intake between any of the OVX groups.

**Table 2:** Body Weight and Composition, and Bone Densitometry of the Whole Body and Spine after 8 Weeks on Dietary Treatments.

	Sham	OVX-Control	OVX-Plum	OVX-Apple	OVX-Apricot	OVX-Grape	OVX-Mango
Final Body Weight (*g*)	24.9 ± 0.6^d^	31.2 ± 0.7^a^	26.1 ± 0.9^cd^	26.1 ± 1.0^cd^	29.1 ± 1.2^ab^	29.1± 1.0^ab^	28.2 ± 1.1^bc^
Body Composition							
*Lean Mass (g)*	17.3 ± 0.4	18.7 ± 0.2	18.1 ± 0.5	18.0 ± 0.6	18.6 ± 0.5	18.3 ± 0.5	18.3 ± 0.5
*Fat Mass (g)*	6.2 ± 0.3^c^	11.7 ± 0.6^a^	6.1 ± 0.6^c^	6.3 ± 0.4^c^	9.1 ± 0.7^b^	9.7 ± 0.6^b^	8.7 ± 0.8^b^
*Percent Fat (%)*	26.3 ± 0.6^c^	38.3 ± 1.0^a^	25.0 ± 1.3^c^	25.6 ± 0.9^c^	32.4 ± 1.4^b^	34.5 ± 1.1^b^	31.7 ± 1.6^b^
Bone Densitometry							
*Whole Body*							
*BMD (mg/cm^2^)*	53.8 ± 0.5^a^	47.3 ± 0.4^e^	49.2 ± 0.5^bc^	47.8 ± 0.4^de^	49.8 ± 0.4^b^	48.9 ± 0.5^bcd^	48.3 ± 0.4^cde^
*BMC (mg)*	564.1 ± 15.2^a^	414.9 ± 14.1^f^	513.2 ± 9.0^b^	481.9 ± 10.0^c^	480.2 ± 9.7^cd^	445.2 ± 9.5^ef^	450.4 ± 5.9^de^
*BMA (cm^2^)*	10.5 ± 0.2^a^	8.8 ± 0.3^e^	10.4 ± 0.2^a^	10.1 ± 0.2^ab^	9.6 ± 0.2^bc^	9.1 ± 0.2^de^	9.3 ± 0.1^cd^
*Spine*							
*BMD (mg/cm^2^)*	56.7 ± 1.6^a^	44.1 ± 1.0^c^	48.4 ± 0.8^b^	46.0 ± 0.9^bc^	47.35 ± 0.6^b^	48.0 ± 1.3^b^	45.6 ± 1.0^bc^
*BMC (mg)*	17.0 ± 0.6^a^	13.0 ± 0.6^cd^	14.5 ± 0.2^b^	12.3 ± 0.4^d^	13.6 ± 0.3^bc^	13.3 ± 0.3^cd^	13.0 ± 0.4^cd^
*BMA (cm^2^)*	0.30 ± 0.01^a^	0.30 ± 0.01^a^	0.30 ± 0.01^a^	0.27 ± 0.01^c^	0.29 ± 0.01^ab^	0.28 ± 0.01^bc^	0.29 ± 0.01^abc^

Final body weight, body composition, and dual energy x-ray absorptiometry (DXA) scans of the whole body and spine after 8 weeks of sham-operated (Sham) and ovariectomized (OVX) mice fed control diet or OVX receiving control diet supplemented with either 25% (w/w) dried plum, apple, apricot, grape, or mango. Values are means ± SE, *n*  =  8 mice in each group. Within a given row, groups that share the same superscript letter are not significantly different (*p*<0.05) from each other.

The reduction in uterine weights in all OVX groups confirmed ovarian hormone deficiency was achieved with surgery and the absence of any estrogenic effects (*i.e*., increase in uterine weight) associated with any of the dietary regimens (*data not shown*).

### Bone Mineral Area, Content and Density

Two weeks post-surgery, DXA analysis confirmed a decrease (*p* < 0.05) in whole body (Sham  =  49.8 ± 0.5 mg/ cm^2^; OVX  =  48.0 ± 0.6 mg/ cm^2^) and spine (Sham  =  47.3 ± 0.8 mg/ cm^2^; OVX  =  43.9 ± 0.8 mg/ cm^2^) BMD in the OVX compared to the Sham baseline groups. After 8 weeks of treatment, the groups consuming the dried plum, apricot and grape supplemented diets had a higher whole body and spine BMD compared to the OVX cohort (**Table 2**). These improvements in whole body and spine BMD with dried plum were a result of increased BMC compared to the OVX animals on control diet. In contrast, the animals receiving the apricot supplemented diet exhibited an increase in whole body BMC, but to a lesser extent than that observed in the dried plum group. The increase in whole body and spine BMD in the group receiving the grape supplemented diet was not due to any statistically significant alterations in BMC compared to the OVX control animals.

### Trabecular and Cortical Bone Microarchitecture

**Figure 1:**
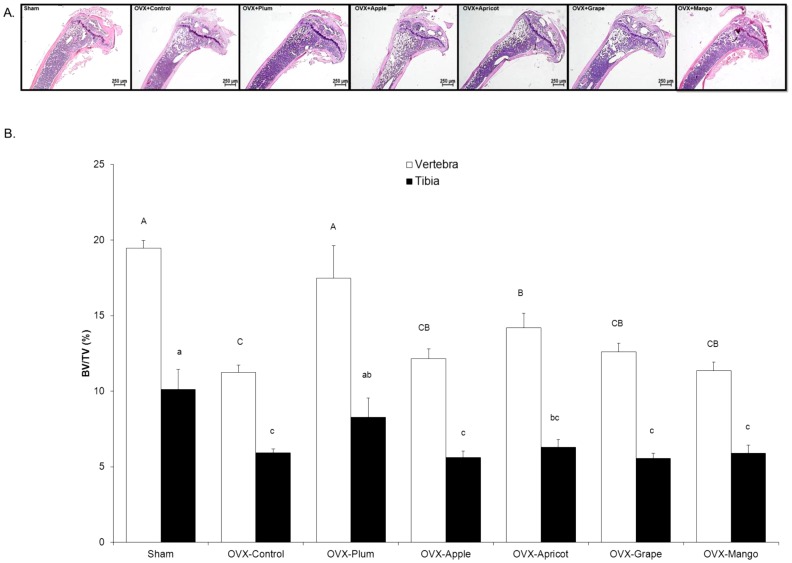
Histological Images of the Tibia and µCT Analyses of Trabecular Bone Volume in the Vertebra and Tibia. Representative images of the proximal tibia are shown with H&E stain following the 8 week treatment period (A). Comparisons of trabecular bone morphometric parameters in sham-operated (Sham) and osteopenic ovariectomized (OVX) mice fed control diet, or control diet supplemented with either 25% (w/w) dried plum, apple, apricot, grape, or mango. Trabecular bone microarchitectural of (B) bone volume/total volume (BV/TV) in the vertebral body (□) and proximal tibia (▪). Bars represent the mean ± SE, *n*  =  6 mice in each group. Bars that share the same superscript letter are not significantly different from each other (*p<*0.05).

A representative micrograph from each treatment group is shown to depict gross-morphological alterations occurring in the proximal tibia after 8 weeks of dietary treatment (**Figure 1A**). To further examine the changes in the trabecular and cortical bone compartments of the tibia and lumbar vertebra, µCT scans of the proximal tibial metaphysis and the fourth lumbar vertebra were analyzed. At baseline, trabecular bone BV/TV tended to decrease in the proximal tibial metaphysis (Sham  =  11.0 ± 0.5%, OVX  =  8.8 ± 0.1%; *p = 0*.0627) and vertebral body (Sham  =  12.7 ± 0.7%, OVX  =  10.9 ± 0.6%; *p = *0.0521), which is similar to previous reports in our laboratory with OVX-induced bone loss [30]. No alterations were observed in cortical bone at baseline (*data not shown*).

**Figure 2:**
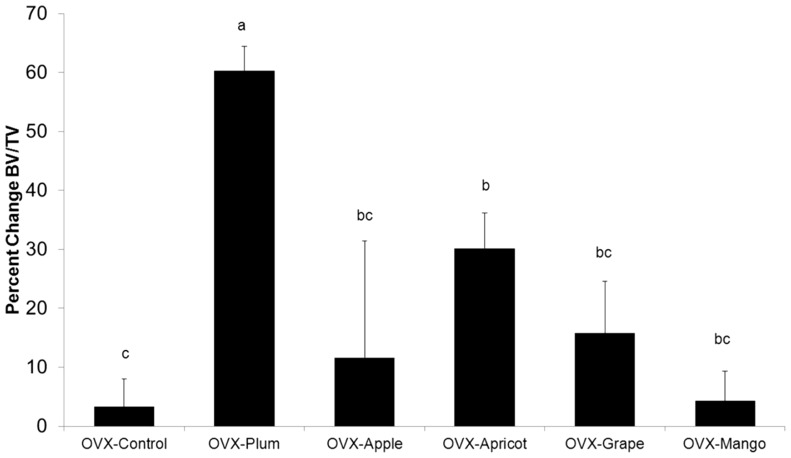
Percent Change in Vertebral Trabecular Bone Normalized to OVX-Control at Baseline. Percent change in vertebral BV/TV in ovariectomized (OVX) mice fed control diet, or control diet supplemented with either 25% (w/w) dried plum, apple, apricot, grape, or mango relative to baseline OVX controls. The percent change was calculated by determining the difference between the OVX group mean BV/TV at baseline and BV/TV of individual animals at the final time point, and then expressing that value relative to baseline. Bars represent the mean ± SE, *n*  =  6 mice in each group. Bars that share the same superscript letter are not significantly different from each other (*p<*0.05).

By the end of the 8 week dietary treatment period, the OVX animals on control diet had experienced an approximately 40% loss of trabecular bone (*i.e*., BV/TV) at both the vertebral and tibial sites (**Figure 1B**). While the dried plum and apricot groups had a higher vertebral BV/TV (**Figure 1B** **)** and Tb.N. (**Table 3**) than the OVX control group (*p<*0.05), the only animals that had a similar BV/TV to the Sham were the mice on the dried plum diet. Based on comparisons to the OVX animals at baseline, dried plum had an anabolic effect on trabecular bone at the vertebral site (**Figure 2**) but only prevented bone loss at the proximal tibia. Vertebral Tb.Th. also increased in the dried plum group, an effect that was not observed with the other dietary treatments (**Table 3**). At the tibial site, dried plum was the only dietary treatment in which BV/TV (**Figure 1B** **)** and Tb.N. (**Table 3** **)** were comparable to the Sham animals. While no dietary treatment was able to reverse the OVX increase in tibial Tb.Sp., dried plum was similar to both the OVX control and Sham control groups (**Table 3**).

**Table 3:** Bone Microarchitectural Parameters of the Lumbar Vertebra and Tibia.

	Sham	OVX-Control	OVX-Plum	OVX-Apple	OVX-Apricot	OVX-Grape	OVX-Mango
**Vertebral Body**							
*Tb.N. (1/mm)*	3.95 ± 0.06^a^	2.96 ± 0.11^c^	3.99 ± 0.26^a^	3.08 ± 0.14^bc^	3.50 ± 0.18^b^	3.14 ± 0.11^bc^	2.99 ± 0.10^c^
*Tb.Th. (mm)*	0.049 ± 0.001^a^	0.038 ± 0.001^c^	0.043 ± 0.002^b^	0.039 ± 0.001^c^	0.040 ± 0.001^bc^	0.040 ± 0.001^bc^	0.038 ± 0.001^c^
*Tb.Sp.(mm)*	0.20 ± 0.01^d^	0.30 ± 0.01^a^	0.21 ± 0.02^cd^	0.29 ± 0.02^a^	0.25 ± 0.02^bc^	0.28 ± 0.01^ab^	0.30 ± 0.01^a^
*ConnDens (1/mm^3^)*	119.5 ± 6.7^a^	85.1 ± 6.9^b^	137.2 ±10.6^a^	81.0 ± 6.3^b^	119.4 ± 8.9^a^	91.8 ± 6.8^b^	88.8 ± 5.5^b^
*SMI*	1.2 ± 0.1^c^	1.8 ± 0.1^a^	1.3 ± 0.2^c^	1.7 ± 0.1^ab^	1.5 ± 0.1^bc^	1.6 ± 0.1^ab^	1.8 ± 0.1^a^
*Tb.Density (mg/cm^3^)*	299.4 ± 5.0^a^	208.35 ± 11.2^c^	282.9 ± 20.8^a^	227.4 ± 7.0^bc^	247.4 ± 9.6^b^	234.1 ± 6.2^bc^	223.1 ± 13.2^bc^
**Proximal Tibial Metaphysis**							
*Tb.N. (1/mm)*	2.91 ± 0.29^a^	1.86 ± 0.11^b^	2.20 ± 0.23^a^	1.75 ± 0.13^b^	1.92 ± 0.16^b^	1.72 ± 0.08^b^	1.74 ± 0.15^b^
*Tb.Th. (mm)*	0.034 ± 0.001^a^	0.032 ± 0.001^b^	0.033 ± 0.001^ab^	0.032 ± 0.001^ab^	0.033 ± 0.001^ab^	0.032 ± 0.001^ab^	0.034 ± 0.001^ab^
*Tb.Sp.(mm)*	0.32 ± 0.03^c^	0.51 ± 0.03^ab^	0.44 ± 0.05^bc^	0.55 ± 0.05^a^	0.50 ± 0.05^ab^	0.55 ± 0.24^a^	0.56 ± 0.05^a^
*ConnDens (1/mm^3^)*	69.2 ± 7.9^a^	32.2 ± 5.1^bc^	43.8 ± 13.2^b^	25.7 ± 4.2^bc^	30.8 ± 7.5^bc^	15.9 ± 1.2^c^	30.3 ± 4.8^bc^
*SMI*	1.8 ± 0.2^b^	2.4 ± 0.1^a^	2.1 ± 0.1^ab^	2.3 ± 0.1^a^	2.2 ± 0.1^a^	2.34 ± 0.1^a^	2.27 ± 0.03^a^
*Tb.Density (mg/cm^3^)*	137.3 ± 16.1^a^	80.9 ± 9.6^bc^	104.0 ± 17.8^b^	66.8 ± 5.5^c^	76.7 ± 7.2^bc^	67.8 ± 4.3^c^	72.0 ± 7.0^c^
**Tibia Diaphysis**							
*Cort.Th. (mm)*	0.22 ± 0.04^a^	0.20 ± 0.03^bc^	0.21 ± 0.01^ac^	0.20 ± 0.01^b^	0.20 ± 0.01^b^	0.20 ± 0.01^b^	0.21 ± 0.01^b^
*Cort. Area (mm^2^)*	0.62 ± 0.01^a^	0.59 ± 0.01^ab^	0.60 ± 0.02^ac^	0.55 ± 0.01^b^	0.58 ± 0.01^ab^	0.54 ± 0.02^b^	0.55 ± 0.01^bc^
*Cort.Med. Area (mm^2^)*	0.86 ± 0.05	0.86 ± 0.04	0.89 ± 0.11	0.83 ± 0.01	0.84 ± 0.02	0.80 ± 0.03	0.75 ± 0.04
*Porosity (%)*	2.78 ± 0.1	2.99 ± 0.1	2.96 ± 0.1	3.14± 0.1	2.87 ± 0.05	2.84 ± 0.1	3.02 ± 0.1

Trabecular bone microarchitectural parameters include: bone volume/total volume trabecular number (Tb.N.), trabecular thickness (Tb.Th.), trabecular separation (Tb.Sp.), connectivity density (ConnsDens), structural model index (SMI), and trabecular density (Tb.Density) of the spine and proximal tibia. Cortical bone includes cortical thickness (Cort.Th.), cortical area (Cort. Area), medullary area (Cort.Med. Area) and cortical porosity of the of the tibial mid-diaphysis from sham-operated (Sham) and ovariectomized (OVX) mice fed control diet or OVX receiving control diet supplemented with either 25% (w/w) dried plum, apple, apricot, grape, or mango for 8 weeks. Values are means ± SE, *n*  =  8 mice in each group. Within a given row, groups that share the same superscript letter are not significantly different (*p*<0.05) from each other.

Consistent with the other µCT data reported in the vertebra, non-morphometric parameters revealed an OVX-dependent compromise in trabecular bone connectivity density, SMI, and density that both the dried plum and apricot diets were able to restore after 8 weeks (**Table 2**). In the tibia, only an OVX effect was observed in connectivity density and apparent mean/ density; however, dried plum had an intermediate effect on SMI.

Analysis of cortical bone parameters of the tibial mid diaphysis revealed a reduced cortical thickness (*p<*0.05) in all OVX animals, regardless of the dietary regimen (**Table 3**). No difference was observed in cortical area between any of the OVX groups. No effect of OVX or dietary treatments was observed on medullary area after 8 weeks.

### Biomechanical Properties of Trabecular Bone

Finite element (FE) analysis revealed decreased total force, stiffness, and size independent stiffness in the trabecular bone of the vertebral body of the OVX control animals compared to Sham (**Table 4**). However, all vertebral biomechanical parameters of the OVX groups receiving dried plum and apricot diets were completely reversed to the level of the Sham animals (**Table 4**). Dried grape was able to improve total force values compared to OVX control diet, however, not to the level of Sham (**Table 4**). Although a trend in total force (*p* = 0.0571) and stiffness (*p* = 0.0554) was observed in the tibia between groups, the only significant treatment effect was an increase in size independent stiffness in the dried plum group compared to all other groups (**Table 4**).

**Table 4:** Biomechanical Properties of Trabecular Bone in the Lumbar Vertebra and Proximal Tibia.

	Sham	OVX-Control	OVX-Plum	OVX-Apple	OVX-Apricot	OVX-Grape	OVX-Mango
**Vertebra**							
*Total Force (N)*	1.6 ± 0.2^a^	0.6 ± 0.1^b^	1.7 ± 0.4^a^	0.9 ± 0.1^bc^	1.2 ± 0.2^ac^	1.1 ± 0.1^bc^	0.6 ± 0.1^b^
*Stiffness (1×10^3^N/m)*	273.1 ± 32.0^ac^	113.6 ± 14.7^b^	308.2 ± 68.4^a^	166.5± 16.3^bd^	212.6 ± 26.0^cd^	187.2 ± 20.9^bc^	112.2 ± 11.5^b^
*Size Independent Stiffness (N/m)*	4.86 ± 0.52^a^	1.9 ± 0.2^c^	4.9 ± 1.1^a^	2.7 ± 0.2^bc^	3.4 ± 0.5^ab^	3.0 ± 0.3^bc^	1.8 ± 0.2^bc^
**Tibia**							
*Total Force (N)*	0.60 ± 0.24	0.46 ± 0.12	1.25 ± 0.41	0.39 ± 0.08	0.37 ± 0.17	0.36 ± 0.13	0.32 ± 0.16
*Stiffness (1×10^3^N/m)*	271.6 ± 107.2	209.9 ± 51.8	562.0 ± 184.1	175.1 ± 36.6	168.8 ± 75.0	161.0 ± 59.8	142.9 ± 71.8
*Size Independent Stiffness (N/m)*	0.94 ± 0.36^b^	0.64 ± 0.15^b^	2.14 ± 0.71^a^	0.63 ± 0.14^b^	0.62 ± 0.29^b^	0.63 ± 0.22^b^	0.55 ± 0.29^b^

Finite element (FE) analysis of trabecular bone in the lumbar vertebra and proximal tibial metaphysis of sham-operated (Sham) and ovariectomized (OVX) mice fed control diet or OVX receiving control diet supplemented with either 25% (w/w) dried plum, apple, apricot, grape, or mango for 8 weeks. Values are means ± SE, *n*  =  6 mice in each group. Within a given row, groups that share the same superscript letter are not significantly different (*p*<0.05) from each other.

### Plasma GPx Activity

**Figure 3:**
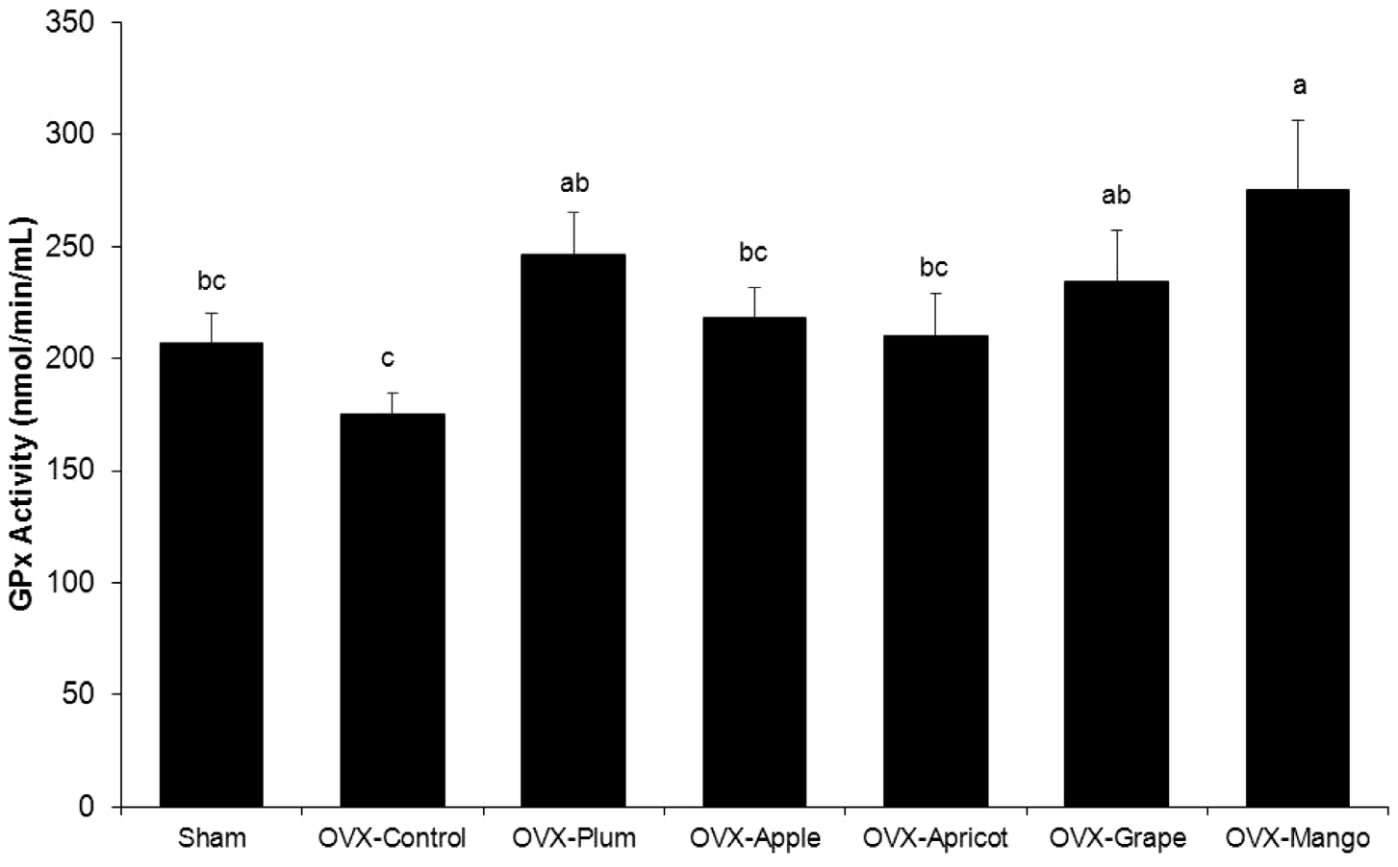
Alterations in Plasma Glutathione Peroxidase Activity (GPx) with Dried Fruit Treatments. Comparisons of plasma GPx in sham-operated (Sham) and ovariectomized (OVX) mice fed control diet, or control diet supplemented with either 25% (w/w) dried plum, apple, apricot, grape, or mango for 8 weeks. Bars represent the mean ± SE, *n*  =  8 mice in each group. Bars that share the same superscript letter are not significantly different from each other (*p<*0.05).

Evaluation of systemic antioxidant activity revealed that the Sham and OVX control groups had similar plasma GPx activity, but the dried plum, grape, and mango groups significantly increased GPx activity compared to the OVX control (**Figure 3**). The apple and apricot diets appeared to have minimal to no impact on plasma antioxidant status compared to animals the groups receiving the control diet (**Figure 3**).

### Osteoblast and Osteoclast Differentiation, Activity and Apoptosis

**Figure 4:**
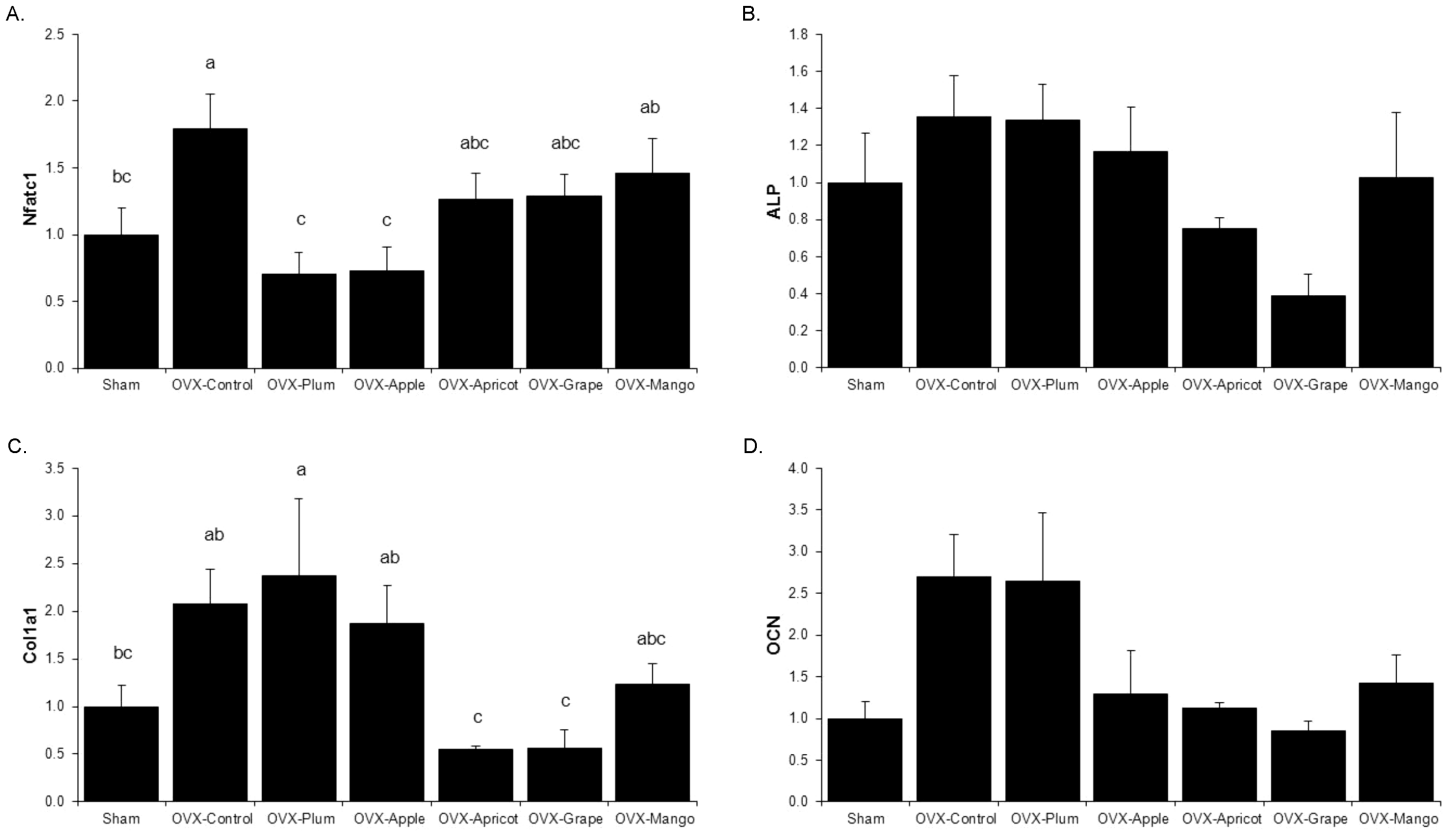
qRT-PCR Analyses of Genes Involved in Osteoclastogenesis and Osteoblast Activity. Bone marrow (A) was analyzed for relative mRNA abundance of the osteoclastgensis gene nuclear factor of activated T-cells (*Nfatc1*), while the flushed femur (B-D) was used to assess genes involved in osteoblast activity and function: alkaline phosphatase (*Alp*), type 1 collagen (*Col1a1*), and osteocalcin (*Ocn*) in sham-operated (Sham) and ovariectomized (OVX) mice fed control diet, or control diet supplemented with either 25% (w/w) dried plum, apple, apricot, grape, or mango. Bars represent the mean ± SE, *n*  =  6 mice in each group. Bars that share the same superscript letter are not significantly different from each other (*p<*0.05).

The relative abundance of mRNA encoding proteins associated with osteoclast differentiation and activity were assessed using total RNA prepared from bone marrow aspirates. The abundance of the mRNA encoding the osteoclastogenesis regulator, nuclear factor of activator T-cells cytoplasmic 1 (*Nfatc1*), was up-regulated in OVX control animals as would be expected compared to the Sham. The dried plum and apple diets suppressed the OVX-induced increase in *Nfatc1* and restored the relative level of expression to that of the Sham group (**Figure 4A**). No alterations were observed in mRNA abundance of receptor activator for NF- κB ligand (*Rankl*) or osteoprotegerin (*Opg*) (*data not shown*).

To determine alterations in osteoblast activity, RNA from flushed femurs was used to assess gene expression. Type I collagen (*Colla1*) gene expression was increased by OVX and remained elevated in the dried plum, apple and mango groups (**Figure 4C**). No statistically significant alterations were detected in alkaline phosphatase (*Alp*) or osteocalcin (*Ocn*) due to OVX or dietary treatments at the end of the study, which was indicative of no transcriptional regulation of osteoblast activity or mineralization (**Figure 4B and 4D**).

**Figure 5:**
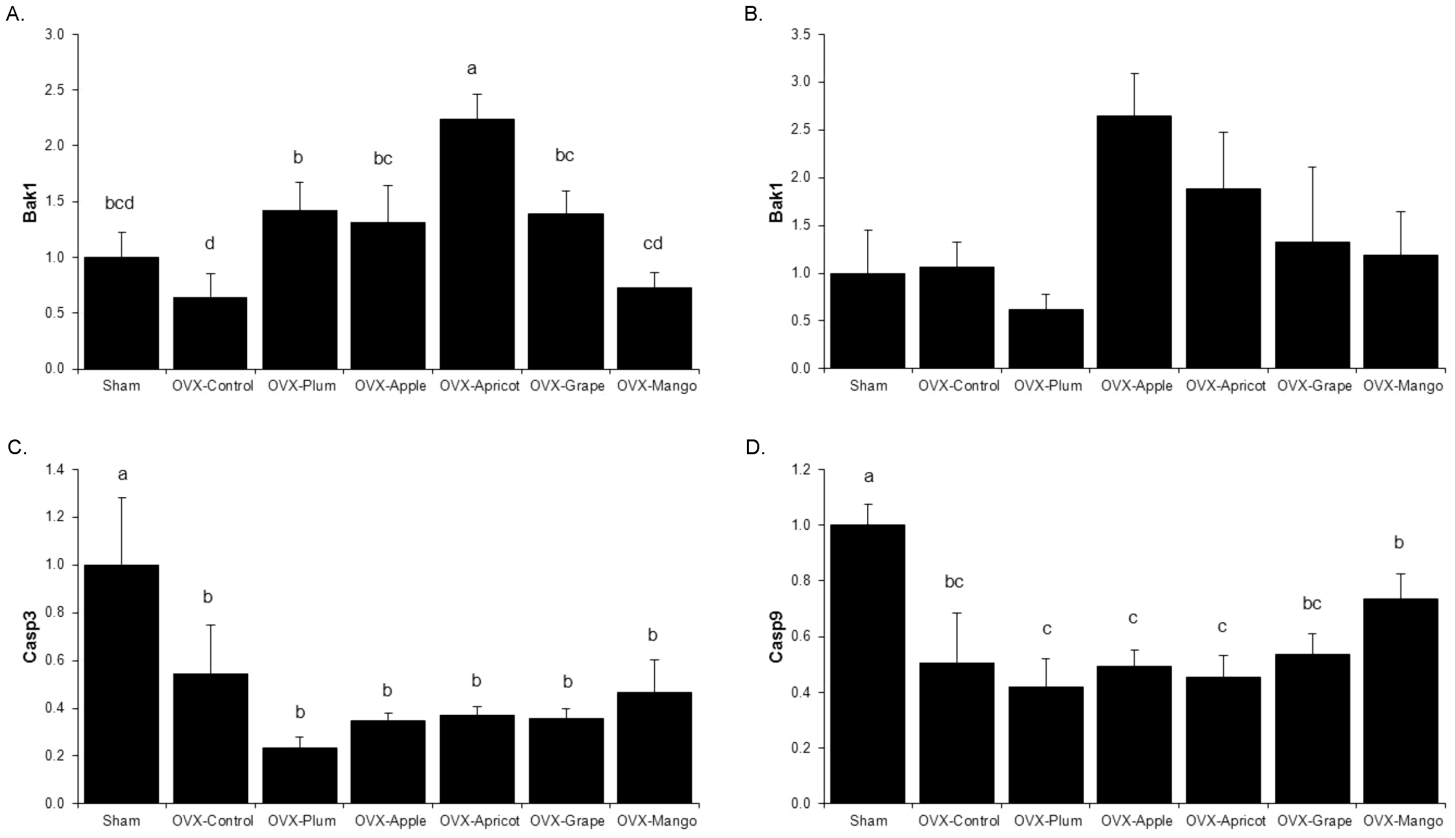
Relative mRNA Abundance of Apoptotic Genes. The relative mRNA abundance of apoptotic genes was assessed in the bone marrow (A) and flushed femur (B–D). Genes of interest including, B-cell lymphoma homologous antagonist/killer (*Bak1*), caspase -3 (*Casp3*) and -9 (*Casp9*) from sham-operated (Sham) and ovariectomized (OVX) mice fed control diet, or control diet supplemented with either 25% (w/w) dried plum, apple, apricot, grape, or mango. Bars represent the mean ± SE, *n*  =  6 mice in each group. Bars that share the same superscript letter are not significantly different from each other (*p<*0.05).

Another plausible mechanism by which the dietary treatments altered bone formation and resorption in the OVX model was by regulating osteoblast and osteoclast apoptosis. In the osteoclast and osteoclast precursor rich bone marrow, Bcl-2 homologous antagonist/ killer 1 (*Bak1*), which promotes apoptosis by forming channels in outer mitochondrial membrane, mRNA abundance was up-regulated by all of the dried fruits except mango, compared to the levels of OVX control animals (**Figure 5A**). In hard tissue (*i.e*., flushed femur), the essential proteases involved in the apoptotic process, caspase-9 (*Casp9*) and its downstream target caspase-3 (*Casp3*), gene expression was down-regulated in response to OVX, but no dietary treatment altered this response (**Figure 5C and 5D**).

## Discussion

The bone densitometry data confirm that dried plum, and to a lesser extent apricot and grape, have the ability to protect bone in osteopenic OVX C57BL/6 mice. Evaluation of the bone microarchitectural parameters revealed that dried plum prevented the loss of trabecular bone in the proximal tibia and was the only fruit to exert an anabolic effect on the trabecular bone of the vertebra. These alterations in the vertebral trabecular bone of animals receiving dried plum coincided with improved biomechanical properties, including bone strength and stiffness. These improvements in the biomechanical and structural properties suggest that dried plum supplementation results in enhanced bone quality.

Due to the role of oxidative stress in bone loss resulting from estrogen deficiency and reports by others that natural compounds in foods can increase plasma GPx in conjunction with improved bone indices, GPx activity was assessed [27]–[29]. The groups supplemented with dried plum, grape and mango groups experienced an increase plasma GPx activity compared to the OVX-control group; however, the lack of a skeletal response in the mango group suggests the favorable effects of dried plum on bone are not solely dependent on GPx antioxidant activity. Our results do not rule out that other antioxidant systems (*e.g.,* catalase activity and superoxide dismutase) altered in response to dried fruit consumption are responsible for the positive effects observed on bone, but these systems warrant further investigation before conclusions regarding the role of antioxidant systems in this context can be made [26], [44].

Due to the apparent novelty of dried plum to not only prevent catabolic activity but also have anabolic effects on bone at some skeletal sites, the relative mRNA abundance of key genes involved in the regulation osteoblast and osteoclast differentiation and activity were assessed. Dried plum decreased osteoclastogenesis through the attenuation of *Nfatc1* gene expression, while simultaneously up-regulating osteoblast activity by enhancing *Col1a1* mRNA. This data is consistent with previous reports of dried plum’s dual anti-resorptive and anabolic actions which represents an uncoupling of bone turnover in favor of accrual of new bone tissue [30], [35]. Although *Rankl* expression was not altered in this study, NFATc1 is the key transcription factor that regulates osteoclastogenesis and is a downstream target of RANK-RANKL signaling. Taken together, these data suggest that dried plum did not regulate *Rankl* at a transcriptional level in this study. Interestingly, dried apple also decreased *Nfatc1* and *Col1a1* transcription, but these alterations in mRNA were not accompanied with beneficial effects on BMD or trabecular bone volume at this time point. These findings raise the issue that dried plum has additional effects, perhaps in part tied to its influence on antioxidant systems that ultimately lead to its unique ability to restore bone

The concept that dried plum and other fruits could be altering osteoblast and osteoclast via apoptosis was also explored in this study. Our data show that OVX decreased *Bak1* gene expression in the bone marrow compared to Sham, suggesting estrogen withdrawal not only promotes osteoclastogenesis by up-regulating *Nfatc1* but also attempts to suppress mitochondria-mediated apoptosis. Dried plum, apple, apricot, and grape prevented the OVX-induced decrease in *Bak1* gene expression, which indicates that the dried plum and apple groups not only suppressed the formation of osteoclasts but may prohibit the OVX-induced decrease in apoptosis. Conversely, in the hard tissue there were no effects of dietary treatments on the relative abundance of *Casp3* and *-9* mRNA in the OVX animals which is consistent with a decrease in osteoblast apoptosis [28]. While these findings provide some evidence of apoptotic regulation by dried plum in the current study, the discrepancies between the early apoptosis marker *Bak1* versus the later apoptosis markers, *Casp9* and *Casp3,* suggest a possible difference in transcriptional regulation of apoptosis in bone marrow and mineralized bone.

Dried plum’s ability to both prevent bone loss and restore bone has been postulated to be due to the high polyphenol content or perhaps the unique polyphenolic compound profile acting as antioxidant and/or anti-inflammatory agent [34]. A recent report from our laboratory showed that an extract of the total polyphenols from dried plum reduced osteoclastogenesis and up-regulated osteoblast differentiation and activity, both under normal and inflammatory conditions [35], [36]. Thus it stands to reason that fruits with similar polyphenolic profiles might be able to exert comparable actions on the skeleton. Dried plum (‘Improved French’) is rich in hydroxycinnamic acids including, neochlorogenic, cryptochlorogenic, and chlorogenic acid [45], [46]. Of the fruits tested in the present study, a large portion of apricot’s polyphenolic compounds are made up of neochlorogenic and chlorogenic acid, similar to dried plum [45], [47]. Although apricot and, to a lesser extent, apple have similar polyphenolic compounds, dried plum is unique in its combination and overall content of the polyphenols [45], [48]. Additionally, dried plums are a good source of other nutrients with known effects on bone, including oligofructose, magnesium, vitamin K, boron and potassium [49], [50]. This study suggests that dried plum may represent a unique combination of nutrients and polyphenolic compounds that is responsible for the observed anabolic activity of the skeleton. However, further investigation of the bioactive components is needed and it is premature to draw any specific conclusions at this time.

In summary, this is the first study to demonstrate the extent to which dried plum restores bone in osteopenic OVX animals compared to apple, apricot, grape and mango supplementation. The potency of dried plum was unique in the sense that it not only prevented bone loss, but also induced an anabolic response in the vertebra. These structural changes coincided with local up-regulation of indicators of osteoblast activity and the down-regulation of osteoclastogenesis. These alternations in mediators of bone metabolism occurred in conjunction with enhanced systemic GPx activity. While this study provides new insight into the alterations in bone metabolism as it relates to dried plum, it is clear that further delineation of these mechanisms is warranted. A limitation of this study is that most of the data presented here provide evidence for transcriptional regulation of osteogenesis and it remains to be observed whether dried plum affects osteoblastogenesis and osteoclastogenesis on a translational level as well. Furthermore, the bioactive component(s) in dried plum remain to be identified and ongoing studies are designed to determine whether it is a single component or the unique combination of nutrients and phytochemicals in dried plum that is responsible for the osteoprotective effects. The continued investigation of how dried plum is able to mediate these effects on bone is important in optimizing this potential dietary strategy for osteoporosis prevention and may provide insight for the development of novel interventions for postmenopausal bone loss.
